# Creation and Initial Validation of the International Dysphagia Diet Standardisation Initiative Functional Diet Scale

**DOI:** 10.1016/j.apmr.2018.01.012

**Published:** 2018-02-08

**Authors:** Catriona M. Steele, Ashwini M. Namasivayam-MacDonald, Brittany T. Guida, Julie A. Cichero, Janice Duivestein, MRSc, Ben Hanson, Peter Lam, Luis F. Riquelme

**Affiliations:** a the Toronto Rehabilitation Institute e University Health Network, Toronto, ON, Canada; b Faculty of Medicine, Rehabilitation Sciences Institute, University of Toronto, Toronto, ON, Canada; c Department of Communication Sciences and Disorders, Adelphi University, Garden City, NY; d International Dysphagia Diet Standardisation Initiative, Brisbane, QLD, Australia; e School of Pharmacy, University of Queensland, Brisbane, QLD, Australia; f School of Clinical Sciences, Queensland University of Technology, Brisbane, QLD, Australia; g Access Community Therapists, Vancouver, BC, Canada; h University of British Columbia, Vancouver, BC, Canada; i University College London, London, UK; j Peter Lam Consulting, Vancouver, BC, Canada; k New York-Presbyterian Brooklyn Methodist Hospital, Brooklyn, NY and; l New York Medical College, Valhalla, NY

**Keywords:** Deglutition, Deglutition disorders, Rehabilitation

## Abstract

**Objective:**

To assess consensual validity, interrater reliability, and criterion validity of the International Dysphagia Diet Standardisation Initiative Functional Diet Scale, a new functional outcome scale intended to capture the severity of oropharyngeal dysphagia, as represented by the degree of diet texture restriction recommended for the patient.

**Design:**

Participants assigned International Dysphagia Diet Standardisation Initiative Functional Diet Scale scores to 16 clinical cases. Consensual validity was measured against reference scores determined by an author reference panel. Interrater reliability was measured overall and across quartile subsets of the dataset. Criterion validity was evaluated versus Functional Oral Intake Scale (FOIS) scores assigned by survey respondents to the same case scenarios. Feedback was requested regarding ease and likelihood of use.

**Setting:**

Web-based survey.

**Participants:**

Respondents (NZ170) from 29 countries.

**Interventions:**

Not applicable.

**Main Outcome Measures:**

Consensual validity (percent agreement and Kendall t), criterion validity (Spearman rank correlation), and interrater reliability (Kendall concordance and intraclass coefficients).

**Results:**

The International Dysphagia Diet Standardisation Initiative Functional Diet Scale showed strong consensual validity, criterion validity, and interrater reliability. Scenarios involving liquid-only diets, transition from nonoral feeding, or trial diet advances in therapy showed the poorest consensus, indicating a need for clear instructions on how to score these situations. The International Dysphagia Diet Standardisation Initiative Functional Diet Scale showed greater sensitivity than the FOIS to specific changes in diet. Most (>70%) respondents indicated enthusiasm for implementing the International Dysphagia Diet Standardisation Initiative Functional Diet Scale.

**Conclusions:**

This initial validation study suggests that the International Dysphagia Diet Standardisation Initiative Functional Diet Scale has strong consensual and criterion validity and can be used reliably by clinicians to capture diet texture restriction and progression in people with dysphagia.

Diet texture modification is the most commonly used intervention for people with dysphagia.^1^ Although the extent of dietary modification may be seen as a proxy measure of dysphagia severity, functional outcome scales for dysphagia are vague on this point. The goal of this study was to conduct preliminary validation of a new scale, designed to capture and communicate the degree of diet texture restriction recommended by clinicians for patients with dysphagia according to the new International Dysphagia Diet Standardisation Initiative (IDDSI) framework.^2^ This new scale is known as the IDDSI Functional Diet Scale.

Table 1 provides an overview of existing functional outcome scales for swallowing. Most commonly, higher scores indicate less severe impairment, consistent with the conventions of the FIM.^12^ Although reference may be made to the extent of diet texture restriction recommended for a patient, these references lack context. Terms like “levels below a regular diet status” imply knowledge of a diet framework with commonly understood levels of consistency; however, no such framework is identified. Around the world, different conventions have been in place with respect to the number of diet texture levels used in dysphagia management and the directionality and terminology for labeling these levels.^13^

Recognition of the lack of a common framework for diet texture classification became the driving impetus behind development of the IDDSI framework,^2^ a new scheme for describing and categorizing foods and drinks according to their texture or flow characteristics. The framework includes 8 levels, organized in 2 intersecting pyramids (fig 1), with the outer levels (0 and 7) representing unmodified drinks and foods and intervening levels representing progressively greater degrees of texture modification. A novel aspect of the IDDSI framework is the overlap zone at levels 3 and 4, in which the characteristics of foods and drinks are equivalent. Internationally, several national professional associations and guidelines bodies (including those in the United States, Canada, and Australia) have formally announced intent to adopt the IDDSI framework.^14-16^

The IDDSI Functional Diet Scale was developed as an accompaniment to the IDDSI framework to capture the degree of diet texture restriction recommended for a patient based on assessment by a qualified clinician. The scale does not indicate the specific textures that are recommended, rather it classifies dysphagia severity according to the degree of diet limitation (ie, the number of levels on the IDDSI framework that a patient can consume). Lower numbered scores on the IDDSI Functional Diet Scale reflect tighter diet texture restriction. The scale captures clinician recommendation rather than the results of a standardized measure of swallowing physiology or function or the actual behavior of the patient, which may or may not be consistent with the clinician's recommendation.

Each level on the IDDSI framework is identified by a descriptive name (eg, mildly thick), a color, and a number. Detailed descriptors and methods for testing foods and drinks to confirm their place in the framework are provided on the IDDSI website (www.iddsi.org). In clinical practice, a modified texture diet order is expected to comprise 2 levels from the IDDSI framework: first the food level and then the drink level. This is consistent with clinical conventions for specifying diets, beginning with the nutritional specification (eg, low sodium), followed by food texture, and terminating with liquid consistency.^17,18^ The IDDSI Functional Diet Scale score is intended as an accompaniment to the diet texture prescription and can be derived using a matrix similar to a mileage chart (fig 2). The IDDSI Functional Diet Scale score corresponds to the number in the intersecting cell of the column showing the food level and the row showing the drink level recommended for the patient. An IDDSI Functional Diet Scale score of 0 applies for recommendations of nothing by mouth (NPO), with exclusive nonoral feeding. Similarly, an IDDSI Functional Diet Scale score of 1 applies when oral intake is restricted to any single level on the IDDSI framework. The specific level(s) recommended cannot be understood from the IDDSI Functional Diet Scale score alone. This is similar to the convention of other functional outcome scales, such as the FIM,^12^ which specifies the degree of assistance or supervision required (eg, minimal, moderate, maximal, total) for an activity (eg, grooming or mobility), without identifying the specific types of assistance provided (eg, wheelchair vs walker). With respect to diet texture modifications, certain combinations of food and drink levels are not allowed on the IDDSI Functional Diet Scale and are marked N/A (not applicable) in figure 2 because they represent errors of logic in the overlap zone of levels 3 and 4. It is not logical to specify a food texture at level 3 (liquidized) while permitting level 4 (extremely thick drinks). Similarly, it is not logical to permit liquidized or pureed foods for patients who are considered unable to tolerate any oral intake of liquids, or to permit moderately or extremely thick liquids for patients who are considered unable to tolerate any oral intake of foods.

An assumption of the IDDSI Functional Diet Scale is that the 2 levels specified in a diet texture prescription bracket a range of food and drink levels that are suitable for the person with dysphagia to consume. For example, figure 3A illustrates a recommendation for level 5 (minced and moist foods) with level 2 (mildly thick liquids); it follows that the clinician would also be comfortable with the patient receiving level 4 (pureed foods/ extremely thick liquids) and level 3 (liquidized foods/moderately thick liquids). The IDDSI Functional Diet Scale score would be 4, indicating that 4 levels on the IDDSI framework (ie, levels 2, 3, 4, and 5) are permitted for the patient. Figure 3B shows a second example: for a recommendation of level 3 (liquidized foods/ moderately thick liquids) and level 1 (slightly thick liquids), the IDDSI Functional Diet Scale score would be 3, capturing the fact that level 2 (mildly thick liquids) would also be allowed.

The purpose of this study was to conduct initial evaluation of the psychometric properties of the IDDSI Functional Diet Scale. The specific scale properties of interest were consensual validity, interrater reliability, and criterion validity. The study aims also included obtaining feedback regarding perceived scale utility, determining the degree of consensus regarding the concept of expressing diet recommendations as a bracketed range of IDDSI levels, and exploring the possible addition of a diacritic (+) to denote therapeutic introduction of food or drink items from a more advanced IDDSI framework level.

## Methods

A Google Survey^a^ was developed and launched on September 1, 2016. Ethics approval was obtained from the local institutional review board. The survey introduction stated clearly that participation was voluntary and responses would remain non-identifying in all reports arising from the project. Participants were free to withhold responses at any stage without penalty. Notices advertising the survey were distributed to dysphagia clinicians via social media and on the IDDSI and principal investigator websites. The survey was organized in 3 sections. The first section was demographic questions regarding the respondent's country of residence, profession, level of education, years of clinical practice with dysphagia, and caseload. The second section was 16 case scenarios (infant through geriatric) in which a diet texture recommendation was specified (see appendix 1 for examples of 10 of these cases). Respondents were asked to review each case scenario and assign both an IDDSI Functional Diet Scale score and a Functional Oral Intake Scale (FOIS) score. These were compared with reference scores previously established by consensus among a subgroup of the authors (C.M.S., A.M.N.-M., L.F.R., and J.D.); this subgroup comprised dysphagia clinicians with 4 to >20 years' experience with acute, rehabilitation, and community-based patients across the age span. The third section was questions requesting input regarding IDDSI Functional Diet Scale scoring rules (5-point Likert scales with comment boxes).

After 3.5 weeks, the 3-day moving average for survey response frequency dwindled to 4. Strong response stability for the IDDSI Functional Diet Scale scoring was shown across quartile batches of the responses received to date. Therefore, a decision was made to close the survey.

### Analysis

Statistical analyses were performed in SPSS version 24.0.^b^ Frequency counts were tabulated for categorical and ordinal responses (demographics and qualitative questions). Consensual validity was measured based on the agreement in IDDSI Functional Diet Scale scores for the 16 case scenarios between the survey responses and the author panel reference scores (percent agreement and Kendall τ). Interrater reliability was calculated across successive quartile batches of the response pool using Kendall concordance (*W*) and intraclass coefficients (ICCs). Criterion validity was measured by comparing the IDDSI Functional Diet Scale scores selected by survey respondents with the corresponding FOIS scores selected for the same case scenarios (Spearman rank correlation analysis).

Qualitative analysis was performed on the comments provided in response to the perceived utility and feedback questions. One team member (B.T.G.) reviewed all of these comments and prepared a thematic coding system. A second team member (A.M.N.-M.) then independently reviewed and coded all comments. A consensus meeting was then held to resolve discrepancies and finalize coding.

## Results

### Survey respondents

**Fig 4:**
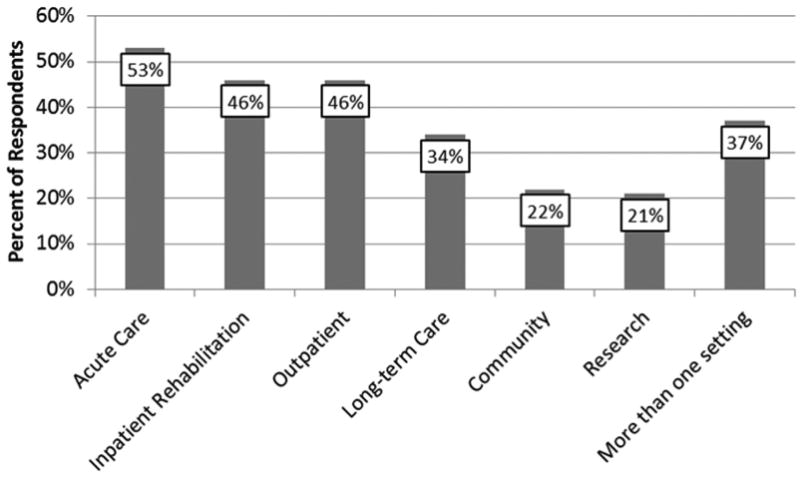
Work settings reported by survey respondents.

In total, 170 responses were received from 29 countries, as summarized in table 2. The professional profile of respondents included speech-language pathologists (80%), dietitians (10%), physicians (7%), and smaller numbers of representatives from other professions, including occupational therapists (n = 2), physical therapist (n=1), dentist (n=1), and food technologist (n=1). Almost half of the respondents (49%) reported having > 10 years of clinical experience, with a further 42% reporting 3 to 10 years of experience. Inquiries regarding caseload revealed that 25.5% of respondents worked with adults, 41.8% worked with seniors, and 6% worked with children. The remaining 26.6% reported working with caseloads of mixed age. Figure 4 illustrates respondents' work settings; slightly more than one third of participants reported working in >1 type of setting.

### Consensual validity

**Fig 5:**
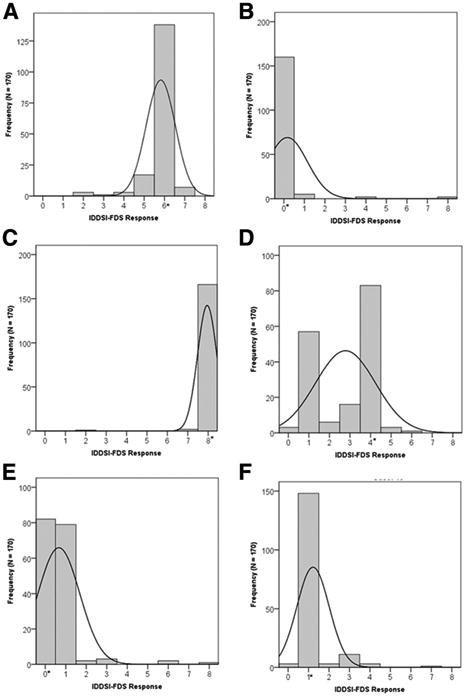
Histograms showing the distributions of IDDSI-FDS scores assigned by survey respondents to 6 examples from the 16 case scenarios used in the study. Expected IDDSI-FDS scores are shown by asterisks. Details for these examples are as follows. (A) Appendix 1, Case 1: Diet texture prescription: level 5 (minced and moist foods) and level 2 (mildly thick drinks). The expected IDDSI-FDS score (ie, 6) was selected by 77% of the survey respondents. (B) Appendix 1, Case 2: Diet texture prescription: NPO (ie, no oral intake of foods or drinks). The expected IDDSI-FDS score (ie, 0) was selected by 90% of the survey respondents. (C) Appendix 1, Case 3: Diet texture: level 7 (regular foods) and level 0 (thin drinks). The expected IDDSI-FDS score (ie, 8) was selected by 97% of the survey respondents. (D) Appendix 1, Case 4: Diet texture prescription: a liquid-only diet spanning level 0 (thin drinks) to level 3 (moderately thick drinks). Given that level 3 also captures a food level on the IDDSI framework, this prescription would correctly be written as level 3 (liquidized foods) and level 0 (thin drinks). The expected IDDSI-FDS score (ie, 4) was selected by 51% of the survey respondents. (E) Appendix 1, Case 5: Diet texture prescription: NPO. The expected IDDSI-FDS score (ie, 0) was selected by 52% of the survey respondents. The finalized IDDSI-FDS scoring instructions capture the additional allowance of ice chips in therapy with a þ diacritic, such that the correct score would be 0þ. (F) Appendix 1, Case 6: Diet texture prescription: no oral intake of foods with level 1 (slightly thick drinks). The expected IDDSI-FDS score (ie, 1) was selected by 87% of the survey respondents. Abbreviation: IDDSI-FDS, IDDSI Functional Diet Scale.

Figure 5 illustrates the distribution of IDDSI Functional Diet Scale scores selected by the survey respondents for 6 of the case scenarios. Overall, the respondents achieved 73% agreement with the author panel reference scores (ρ= .92, Kendall τb= .84). Post hoc exploration showed no differences in the frequency of agreement/ discrepancy with the reference scores as a function of the respondent's years of clinical experience (<1, 1—2, 3—5, 6—10, or >10y; $\chi_{4}^{2}=5.22$; *P*= .27). For most of the case scenarios the distributions show strong consensus and mode scores were selected by ≥77% of respondents. Where consensus was weaker, 3 patterns were observed. For 3 cases (eg, appendix 1, case 8), a broader distribution of scores was seen, with a skew in scores to the left or right of the mode. For 2 cases (eg, appendix 1, case 10), survey response consensus was high but the mode score of 1 differed from the author panel reference score of 0. This appears to reflect respondent uncertainty regarding scoring in cases of primary nonoral feeding where small amounts of oral intake are permitted in a therapeutic context. Finally, 3 cases (eg, appendix 1, cases 4 and 5) showed bimodal distributions; these split opinions are thought to reflect uncertainty regarding scoring for patients requiring primary nonoral nutrition and a lack of familiarity with purely liquid diets.

### Interrater reliability

IDDSI Functional Diet Scale scores showed strong response stability and high interjudge reliability across successive quartile batches of the dataset (n=43 responses per batch). Kendall concordance was *W*=.873 overall, and *W*=.88, *W*=.884, *W*=.896, and *W*=.819, respectively, for the 4 batches. The average ICCs for each batch were .965, .966, .971, and .939, respectively, with the corresponding 95% confidence interval boundaries ranging from .872 to .976.

### Criterion validity

**Fig 6:**
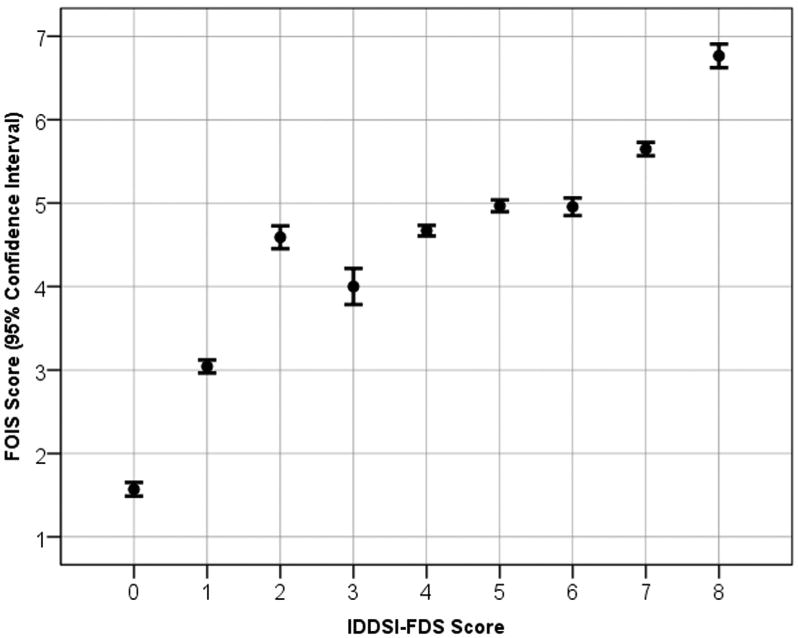
Mapping between survey respondent IDDSI-FDS scores and corresponding FOIS scores for the case scenarios used in the survey. Abbreviation: IDDSI-FDS, IDDSI Functional Diet Scale.

Overall, there was strong correspondence between IDDSI Functional Diet Scale scores and FOIS scores for the case scenarios (Spearman correlation: *R*=.84, *P*<.001). In figure 6, the means and 95% confidence intervals of the FOIS scores that were assigned by respondents to the case scenarios are mapped as a function of the corresponding IDDSI Functional Diet Scale score responses. It can be seen that FOIS scores of 3 to 6 map to a broader range of IDDSI Functional Diet Scale scores (1–7), and FOIS scores clustered between 4 and 5 mapped to an IDDSI Functional Diet Scale range of 2 to 6.

### Questions about perceived IDDSI Functional Diet Scale utility

The number of valid responses on the qualitative section of the survey ranged from 100 to 114; incomplete responses are attributed to the survey being administered exclusively in English.

Respondents indicated general agreement with the bracketed range concept (59% in favor). Slightly more than one quarter (28%) of respondents recommended that tolerance of consistencies between the bracketed boundaries on the IDDSI framework should not be assumed, but confirmed during assessment on a case-by-case basis. There was strong agreement (77%) that the IDDSI Functional Diet Scale score should reflect the main diet recommendation and not reflect therapeutic advancement. Comments from 62% of respondents indicated that therapeutic trials should be annotated separately from diet texture recommendations, and 84% of respondents agreed with the idea of annotating therapeutic advancement with a + diacritic.

## Discussion

It was encouraging to receive survey responses from a wide geographic distribution over a short time frame and to confirm that clinicians around the world with a variety of professional backgrounds found the IDDSI Functional Diet Scale easy to apply to case scenarios describing different diet texture recommendations. The author panelists and the survey respondents showed strong agreement in FOIS scoring (81% in perfect agreement; ICC=.973; 95% confidence interval, .971–.975). This level of agreement on the FOIS is similar to the 85% agreement reported by the scale developers in their original psychometric validation study.^7^ The strong correspondence with FOIS scores shows good criterion validity for the IDDSI Functional Diet Scale. For case scenarios with FOIS scores of 4 and 5, corresponding IDDSI Functional Diet Scale scores spanned a larger range from 2 to 6, suggesting that the IDDSI Functional Diet Scale was better able to capture gradations of diet texture restriction.

The participants in this survey found it straightforward to assign IDDSI Functional Diet Scale scores to most of the case scenarios developed for the validation study. Most of the scenarios with poorer agreement involved a primary recommendation for nonoral nutrition with limited oral intake on a trial or therapeutic basis. Based on the survey responses received in the survey, it has been decided that IDDSI Functional Diet Scale scores will reflect the main diet prescription and that therapeutic diet advances should be annotated using a + diacritic. To illustrate, incorporating this decision into the scoring of appendix 1 case 5 leads to a recommended IDDSI Functional Diet Scale score of 0+, as noted in appendix 1. The + diacritic has the potential to be added to any score on the IDDSI Functional Diet Scale to indicate progress toward tolerance of a greater variety of diet texture levels. For example, if a patient has a prescription for pureed foods and moderately thick liquids (IDDSI Functional Diet Scale score of 2, capturing items at both levels 3 and 4 of the IDDSI framework), several different scenarios might justify annotation with the + diacritic, including (but not limited to) an introduction of mildly thick liquids on a time-limited and closely monitored basis, or the trial introduction of water between meals. The diacritic is simply intended to indicate that some progress away from the specified restriction is being introduced and monitored.

This preliminary validation of the IDDSI Functional Diet Scale explored the ability of clinicians to accurately determine scores based on prespecified diet recommendations. In order for the IDDSI Functional Diet Scale to have true validity to reflect dysphagia severity, it will be necessary to determine whether IDDSI Functional Diet Scale scores vary across groups of patients with different degrees of physiological or functional impairment. A goal for the IDDSI Functional Diet Scale is that it would have broad utility for different patient populations and across different age groups. We are aware of one exploration of this type to date, in a large study of 638 adults residing in long-term care institutions in Canada. In that study, IDDSI Functional Diet Scale scores were derived based on diet orders and compared between residents with and without dysphagia risk (a composite variable determined on the basis of failing a standard dysphagia screening test, signs of coughing during meal observations, and/or prescription of thickened liquids).^19^ IDDSI Functional Diet Scale scores for residents without dysphagia risk ranged from 4 to 8, reflecting an absence of severe diet texture restrictions. The probability of having an IDDSI Functional Diet Scale score<5 was significantly higher in individuals with dysphagia risk.

### Study limitations

A limitation of using social media and web-based communications as a means of inviting survey responses is that the response pool was a voluntary, self-selected convenience sample. In this study, the number of eligible respondents is unknown, as is the number of individuals who became aware of the survey. There was no opportunity to control whether respondents completed the survey independently or in consultation with colleagues. Given that 80% of the responses came from speech-language pathologists, it cannot be assumed that the response patterns are representative of all professions involved in dysphagia management. The sample sizes of professional subgroups were not large enough to allow comparisons by profession. Future studies should engage purposively sampled participants from a variety of professions and health settings.

The design of the case studies was skewed such that one third involved nonoral diets, or transition from nonoral feeding. Notably, these were also the cases where the greatest discrepancy in scoring was seen. A larger pool of cases, balanced for variety of diet and liquids recommendations, may demonstrate even better validity and interrater reliability than seen in this preliminary study. Importantly, the qualitative questions in this study provided guidance regarding scoring instructions for nonoral diets and therapeutic introduction of limited oral intake.

## Conclusions

In this preliminary validation study, the new IDDSI Functional Diet Scale was shown to have strong consensual and criterion validity. A broad sample of 170 clinicians from 29 countries showed that it is straightforward to reliably determine IDDSI Functional Diet Scale scores and that they perceived the scale to have good utility for capturing the degree of diet restriction associated with typical diet combinations used in clinical practice across the age spectrum. The IDDSI Functional Diet Scale captures the degree of diet texture restriction recommended for a patient within the context of the 8 levels of food and drink texture in the IDDSI framework and is suitable for use from infant to geriatric populations. The next step in evaluating the validity of the scale will be to apply the scale to data from larger patient samples to confirm whether IDDSI Functional Diet Scale scores based on diet recommendations capture dysphagia severity in different populations in a clinically meaningful way based on standard metrics of physiological impairment.

## Figures and Tables

**Fig 1:**
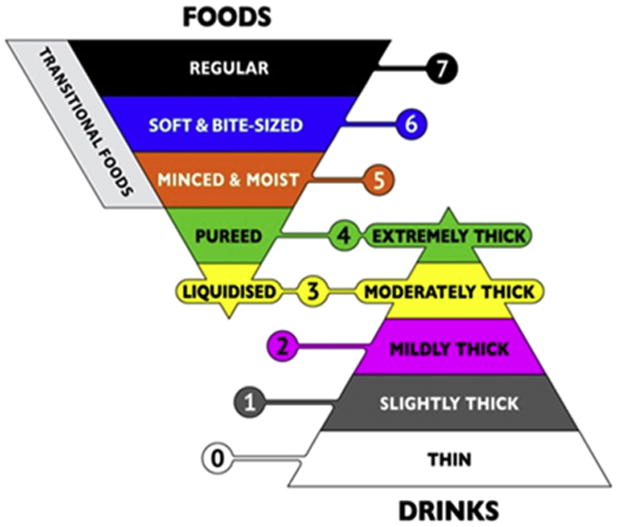
The IDDSI framework.

**Fig 2:**
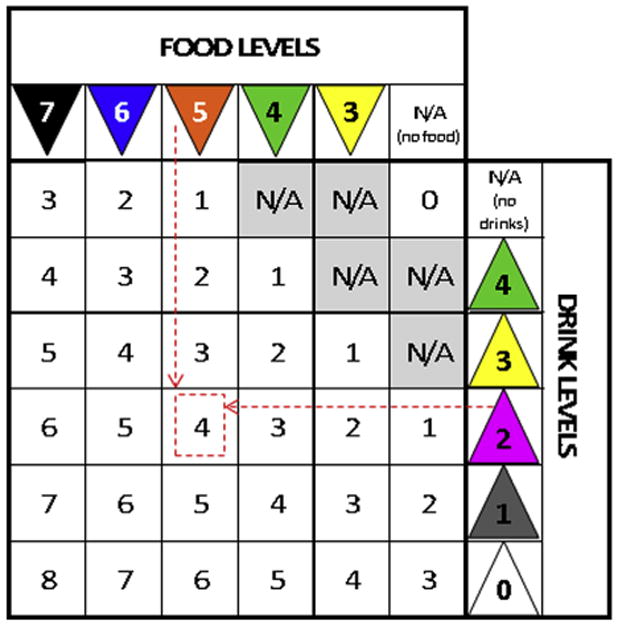
Scoring chart for the IDDSI Functional Diet Scale. To determine the IDDSI-FDS score for a patient, a clinician must find the intersecting cell for the column showing the patient's food texture recommendation and the row showing the patient's drink consistency recommendation. For example, if a patient has a recommendation for a level 5 (minced and moist food texture) and level 2 (mildly thick drinks), the intersecting cell shows an IDDSI Functional Diet Scale score of 4, as indicated by the dashed line arrows and square. Abbreviation: N/A, not applicable.

**Fig 3:**
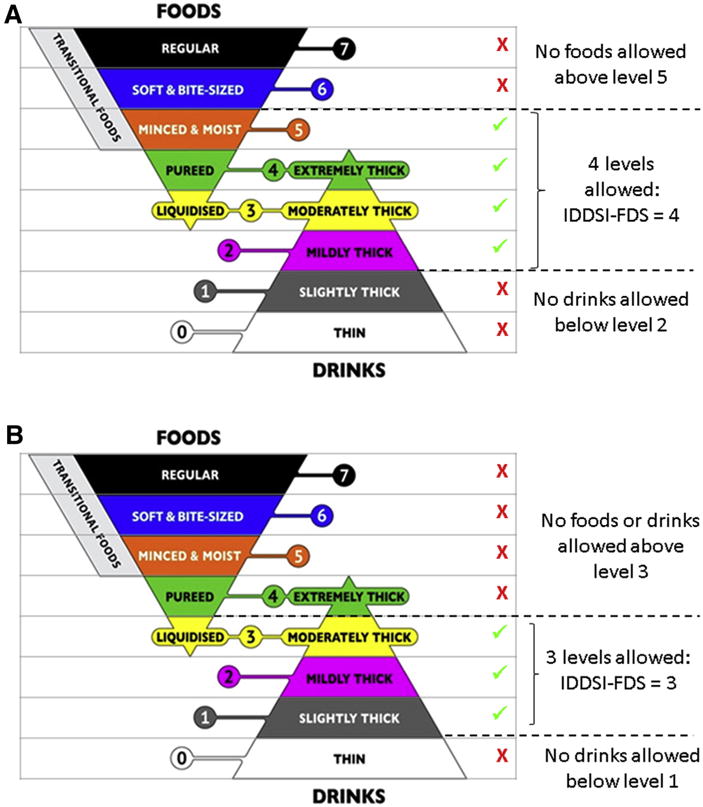
(A) Illustration of IDDSI-FDS score derivation for a diet texture recommendation of level 5 (minced and moist foods) and level 2 (mildly thick liquids). (B) Illustration of IDDSI-FDS score derivation for a diet texture recommendation of level 3 (liquidized foods) and level 1 (slightly thick liquids). Abbreviation: IDDSI-FDS, IDDSI Functional Diet Scale.

**Table 1:** Characteristics of previously published functional outcome scales for swallowing

Scale Name	Target Population	No. of Levels	Direction	Diet Restriction Specifications
Functional Status Scale^3^	Pediatrics	5	1 (normative function) to 5 (severe dysfunction)	Total oral feeding to progressive degrees of assistance, tube-feeding, or parenteral nutrition.
Swallowing Performance Status Scale^4^	General	7	1 (normative function) to 7 (severe dysfunction)	Not described
Dysphagia Outcome and Severity Scale^5^	General	7	7 (normative function) to 1 (severe impairment)	Number of consistencies tolerated or restricted
American Speech-Language Hearing Association National Outcome Measures Scale Functional Communication Measure for Swallowing^6^	General	7	7 (normative function) to 1 (severe impairment)	Number of levels below a regular diet status in either solid or liquid consistency
FOIS^7^	Stroke	7	7 (total oral diet) to 1 (exclusive tube feeding)	Number (single vs multiple) of consistencies taken orally
UK Therapy Outcome Measurement Scale^8,9^	General	6	5 (least severe impairment) to 0 (most severe impairment). Half-point scaling permitted.	Oral vs nonoral nutrition and range of consistencies allowed (limited, modified, most, and full).
Australian Therapy Outcome Measurement Scale^10,11^	General	6	5 (least severe impairment) to 0 (most severe impairment)	Oral vs nonoral nutrition and range of consistencies allowed (limited, modified, most, and full).

**Table 2:** Response frequency by geographic region

Region	Country	Frequency	%
North America (n=67)	United States	36	21.2
	Canada	31	18.2
Europe (n=40)	Ireland	11	6.5
	United Kingdom	6	3.5
	Turkey	4	2.4
	France	3	1.8
	Italy	3	1.8
	Portugal	3	1.8
	Austria	2	1.2
	Germany	2	1.2
	Sweden	2	1.2
	Finland	1	0.6
	The Netherlands	1	0.6
	Norway	1	0.6
	Spain	1	0.6
Oceania (n=30)	Australia	29	17.1
	New Zealand	1	0.6
South America (n=13)	Brazil	11	6.5
	Argentina	1	0.6
	Colombia	1	0.6
Asia (n=13)	Japan	6	3.5
	India	2	1.2
	Singapore	2	1.2
	Iran	1	0.6
	Philippines	1	0.6
	Thailand	1	0.6
Africa (n=6)	South Africa	4	2.4
	Algeria	1	0.6
	Egypt	1	0.6
Missing	Missing	1	0.6
Total		170	100.0

## Appendix 1 Case Scenarios

### Case 1

A 60-year-old woman comes to your outpatient swallowing clinic describing a 2-year history of solid foods “getting stuck” in her throat once or twice per week. She is currently eating regular solids at home and is drinking thin liquids without any reported difficulty. During an instrumental swallowing assessment, you determine that thin liquids are traveling through the oropharynx safely and efficiently, but regular solids are causing large amounts of residue, and require 3 to 4 swallows per bolus to get everything down. Soft and bite-sized foods also cause a fair amount of pyriform sinus residue, but minced and moist solids appear to go down safely and efficiently. You decide to temporarily recommend a diet of minced and moist solids with thin liquids, while additional workup in search of a causal factor is found.

- Food prescription: level 5 (minced and moist)
- Drink prescription: level 0 (thin)
- IDDSI Functional Diet Scale score: 6

### Case 2

An 85-year-old man is having severe difficulties swallowing. On assessment, you find the patient is aspirating all food and liquid consistencies, and the chin tuck position does not improve his swallowing safety. The patient also has extremely poor upper esophageal sphincter opening leading to large amounts of residue on all consistencies. He is even unable to swallow his saliva.

- Food prescription: not applicable. No food level is safe. Nonoral feeding would be appropriate.
- Drink prescription: not applicable. No food level is safe. Nonoral feeding would be appropriate.
- IDDSI Functional Diet Scale score: 0

### Case 3

A 25-year-old woman comes to you following a traumatic brain injury. She was having difficulties with her swallowing immediately after her accident, but now reports improvement with no issues. On assessment you find that she is able to safely and efficiently drink all liquid consistencies and all regular textures.

- Food prescription: level 7 (regular)
- Drink prescription: level 0 (thin)
- IDDSI Functional Diet Scale score: 8

### Case 4

A 52-year-old man has a diagnosis of multiple sclerosis and is having difficulty swallowing, which he thinks is mostly caused by fatigue. On evaluation, you determine that he has significant residue with most food textures and even with extremely thick liquids but that he seems to be able to swallow liquids in the thin to moderately thick range without residue. He does not seem to experience any issues of aspiration. You decide to recommend a liquid diet including thin, slightly thick, mildly thick, and moderately thick liquids.

- Food prescription: level 3 (liquidized)
- Drink prescription: level 0 (thin)
- IDDSI Functional Diet Scale score: 4
- Comment: a recommendation for moderately thick liquids implies that level 3 (liquidized foods) is also appropriate for this patient because of the equivalence of texture and flow characteristics for foods and drinks at level 3.

### Case 5

You have been working with a 27-year-old woman who is recovering from a double lung transplant. She has been NPO for 1 month and fed by gastrostomy tube, but medically she is now doing well and the team is keen for her to begin transitioning back to an oral diet. Your clinical assessment suggests that she may not be fully ready to begin oral intake, but is ready to begin practicing swallows with a safe, starter item (eg, ice chips [or in Japan, dysphagia jelly]).

- Food prescription: not applicable. The primary source of nutrition is by gastrostomy tube.
- Drink prescription: not applicable. The primary source of nutrition is by gastrostomy tube.
- IDDSI Functional Diet Scale score: 0+
- Comment: the primary source of nutrition is by gastrostomy tube. The + diacritic reflects the recommendation for trial oral intake of ice chips in a therapeutic context.

### Case 6

You are working with a mother of a baby who has been having difficulty tolerating thin liquids without aspiration. You determine that the baby is able to swallow slightly thick liquids safely, but that if too much thickener is added, the baby has difficulty expressing fluid through the nipple of the bottle and seems to fatigue very quickly.

- Food prescription: not applicable. This baby is not ready for any solid foods.
- Drink prescription: level 1 (slightly thick)
- IDDSI Functional Diet Scale score: 1

### Case 7

A 45-year-old man is referred to you for a follow-up assessment 3 months after discharge from a stroke rehabilitation center. He is on a minced and moist food texture with mildly thick liquids. Assessment shows that he aspirates thin liquids, but slightly thick liquids prove to be safe. With minced and moist food textures, there is quite significant residue in his pharynx. You decide to recommend a diet change to pureed foods and slightly thick liquids.

- Food prescription: level 4 (pureed)
- Drink prescription: level 1 (slightly thick)
- IDDSI Functional Diet Scale score: 4

### Case 8

An 11-year-old child with spastic cerebral palsy has been on your caseload for several years, and has been managing well on a soft and bite-sized diet with mildly thick liquids. The child is moving to a new school, where a lunch program is available. On the soft lunch diet at this school, sandwiches are frequently offered containing things such as egg salad or tuna salad, with the crusts removed. Your reevaluation of this child suggests that they will not be able to tolerate these sandwiches unless they are precut into bite sized pieces.

- Food prescription: level 6 (soft and bite-sized)
- Drink prescription: level 2 (mildly thick)
- IDDSI Functional Diet Scale score: 5
- Comment: note that bread is not permitted on IDDSI level 6 (soft and bite-sized).

### Case 9

You are working with a 7-year-old child with cerebral palsy who has been NPO and on a gastrostomy feeding tube for total nutrition for the last year. In therapy, you have been working on oral feeding skills using foods that dissolve easily in the mouth with minimal chewing, such as arrowroot biscuits and cheese puffs. This has been going well, and you decide to recommend that the child eat some of these items twice a day in addition to their tube feeding.

- Food prescription: not applicable. The primary source of nutrition is by gastrostomy tube.
- Drink prescription: not applicable. The primary source of nutrition is by gastrostomy tube.
- IDDSI Functional Diet Scale score: 0+
- Comment: the primary source of nutrition is by gastrostomy tube. The + diacritic reflects the recommendation for trial oral intake of transitional foods in a therapeutic context.

### Case 10

You have been asked to assess a 56-year-old man who has completed a recent course of radiation therapy with chemotherapy to treat laryngeal cancer. A gastrostomy feeding tube was placed prior to this patient's cancer treatment, and he has been using the feeding tube as his primary source of nutrition. Your assessment shows that he is feeling very unwell and experiencing a great deal of pain at this stage of his recovery secondary to mucositis. He is aspirating thin and slightly thick liquids silently. You decide to recommend that he stay on the gastrostomy tube feeding, but try to swallow small amounts of mildly thick liquid throughout the day as a way of trying to maintain regular swallowing. You recognize that this oral intake will likely not happen every day, depending on how the patient is feeling.

- Food prescription: not applicable. The primary source of food will be by gastrostomy tube.
- Drink Prescription: not applicable. The primary source of nutrition is by gastrostomy tube.
- IDDSI Functional Diet Scale score: 0+
- Comment: the primary source of nutrition is by gastrostomy tube. The + diacritic reflects the recommendation that the patient try to maintain oral intake of mildly thick liquids.

## List of abbreviations

FOIS: Functional Oral Intake Scale
ICC: intraclass coefficient
IDDSI: International Dysphagia Diet Standardisation Initiative
NPO: nothing by mouth
